# Dr. Stroud on the Death of Christ

**Published:** 1848-01-01

**Authors:** 


ART. V.—*A Treatise on the Physical Cause of the Death of Christ, and its Relation to the Principles and Practice of Christianity.* By WILLIAM STROUD, M.D. 1 vol. 8vo. 1847.

IN no efforts for the benefit of humanity are learning and science more usefully employed than in the elucidation and confirmation of the great truths of our holy religion; and it is truly gratifying to see men of other professions and acquirements coming forth from their own beaten path, to assist the efforts of those whose peculiar duty it is to propagate the gospel. Philosophy, as now understood and pursued, can be regarded in no other light than as the handmaid of revelation; for while in the one, truths are unfolded which could not have been discovered by human researches—in the other, we possess the means which the Almighty has given to us for the investigation of all other truths which are placed within the reach of our limited understanding; and as God, in all his works, labours for one grand end—namely, the happiness of his creatures, there cannot be any rivalry or opposition between the truths of revelation and the truths of natural philosophy, but all must unite in raising our conceptions of the eternal majesty, and infinite power and glory of the Supreme. Happily the day is passed when the discoveries of science were regarded as heresies, and philosophers were burnt at the stake; and it is one of the glories of our times, that thought and private judgment are permitted to range freely and fearlessly through the fields of science, as well as in the green pastures of God's word.

While we thus speak of science as the ally of christianity, we by no means urge upon laymen any practice which might wear the appearance of religious quackery. There are men publicly set apart for the high duties of the ministerial office, and nothing should be done to weaken their influence, or to interfere with their work.

It is, however, quite within the province of our secular duty to afford as much light as our own peculiar studies are capable of affording, to aid the divine in his anxious and difficult investigations; and perhaps there is no science so worthy of this alliance, or better calculated to afford this aid than that of medicine.

The divine has to instruct and discipline the soul; but the soul is imprisoned within a body, and depends for much of its energy, and many of its peculiarities, upon the healthy or unhealthy condition of that body, of which it is the province of the physician to take cognizance.

Were we ignorant of the reciprocal influence of mind and matter upon each other, we should be always erring in our estimate of human character; we could have no exact knowledge of the varied, and ever varying states and conditions of the intellectual and moral faculties, as they exhibit themselves in different orders of mind, or even in the same mind at different times. Joy and sorrow, the light and happy state of mind which enables its possessor to look at the brighter side of all things, and that morose and melancholy disposition which casts its black and dismal shadow upon everything within its range, may all depend upon some peculiarity of organization, or some accidental state of our organism; and when religion takes possession of the hearts of men so oppositely constituted, it displays itself in a manner peculiar to each; one is full of joy, and hope, and love, and zeal—while the other is depressed beyond the necessity of his case; he can scarcely hope, and his zeal and love are but faint and cold; and yet, perhaps, the faith of the one is as strong as that of the other, the difference being only a consequence of physical peculiarities. How much of the fanaticism and aceticism which have at various times astounded and disgusted the world, and what amount of the errors and heresies which have polluted it, were owing to the condition of *livers,* we cannot pretend to say; but we may affirm, without a fear of contradiction, and we think that our readers will corroborate our testimony, that every man's moral and mental state is liable to great fluctuations, and those changes, often the most opposite in their character, almost invariably depend upon the condition of the body. A man is not the same being under the influence of different states of his digestive organs; he varies with the pressure of the atmosphere upon the surface of his body; in the morning he may rise with spirits bounding and buoyant, and a mind alert with vigour and activity, yet, before he lies down upon his bed again, by a fit of indigestion, or suppression of bile, he may be reduced to a state of deplorable melancholy, or brought to the very verge of fatuity.

Now, it is in cases of this description that the physician's skill comes in to aid the efforts of the divine. Physic may not throw much light upon controversial theology, but it may afford material benefit to the practical minister of religion, in enabling him to fix the value of those joys and sorrows which affect his flock; to check the presumptive hope which rests alone upon excited feelings, and revive the drooping spirits of those whose melancholy minds will envelope even the tender mercy of God with distressing and depressing gloom. What all the reasoning and most zealous expostulations of the moralist may fail to effect, a blister will often accomplish; and a blue pill, or black draught, will dissipate clouds, and break down doubts, which have effectually resisted a whole phalanx of scriptural and philosophical arguments.

We do not thus speak because we think lightly of the subject, but, on the contrary, because, from its importance, we deem it necessary to the true interests of religion that it should be fully understood. Those who have witnessed the effects of religious, or, we should rather say, pseudo-religious excitement upon a delicate frame, throwing it into hysterical convulsions, and causing it to writhe as if under a supernatural influence, and have known that this riotous state of the nervous system has been deemed a proof of spiritual conversion, and has been the rock upon which the wretched and deluded fanatic has rested his hopes of the divine favour, will not deem any remarks which are calculated to elicit attention to the important connexion subsisting between our bodily and mental states light and trifling; often a jest will tell, where a sober argument will fail to impress; and even if we can, by the semblance of folly, stir up the minds of any of our psychological friends to a careful, philosophical, and scriptural inquiry into the subject, we shall rejoice in being the means of promoting an investigation of such paramount practical importance to thousands and thousands of sincere and anxious souls.

Such we believe to be one of the legitimate duties of the physiologist, and we think no one will dispute his right so far to enter with the divine upon the management of the soul; but can he do more? does his function cease here, or can he bring his science to elucidate the deep and mysterious truths of religion? The attempt has been made; and we confess that we can see no reason why it should not be done, or that the philosopher should not bring his knowledge forth, when by its light difficulties may be cleared up, and truth brought out from the darkness which envelopes it. Truth is invaluable, and any means which we may use to elicit it are legitimate. On a conjectural question which admits of two or more explanations, neither of which is opposed to God's word or the spirit of christianity, it may be a matter of little importance which view we adopt; but if by any means we can discover that one is true and the other false, then are we bound to make a choice, and hold fast to what we conceive is the truth. We may even go further than this, and say, that no one is justified in sitting down in the repose of ignorance, when he may, by an effort of mind, discover the truth; and if this holds good in matters of ordinary interest, it must, *à fortiori*, be a sound reason for using all our appliances of science and knowledge in the investigation of the high and glorious truths of christianity: and that philosopher who, guided by a deep conviction of the value of the scriptural rule of faith, brings his philosophy to bear upon the mysteries of redemption, either to confirm a settled truth, or to clear up a difficulty, is an ally which the theologian may be justly proud of, and one who deserves the thanks of all christian people. It is only vain philosophy, "*science falsely so called*," that we may fear; genuine philosophy is as ardent and sincere in its inquiries after truth as religion can be, and hand-in-hand these two lovely gifts of God may go onward in the work of goodness and beneficence.

Though we say that we can see no reason why the physiologist may not sometimes bring his scientific knowledge to bear upon the great doctrines of christianity, we are not aware that any such attempt was made before that of Dr. Stroud, in his interesting and valuable work upon the "Physical Causes of the Death of Christ;" and we here tender to the learned and pious physician our thanks for his patient investigation of a subject of so much difficulty and interest. His labours we consider truly valuable, and we have no doubt that his peculiar and original views will, when known, be universally adopted by theologians, in the place of the very unsatisfactory and, we may say, unscriptural explanation of this subject which hitherto has been given.

Now, however much the tender-minded christian may be startled by the title and design of the book, we assure him that he will find within nothing repulsive, but, on the contrary, much to cheer his soul, animate his love, and strengthen his faith. It is an attempt to make clear the highest doctrine of our holy religion, and secure it upon the solid rock which was laid for its foundation; on the stability of which the whole superstructure depends for its existence.

The great doctrine which was a stumbling-block to the Jew, and foolishness to the Greek, was the death of the founder and leader of christianity; but could it have been proved to them that he did not actually die from crucifixion, but hung upon the cross merely for form sake, and then died by his own voluntary act, or that his soul was taken from earth by some supernatural means, then much of the odium attached to his death would have been removed. This, however, is never attempted in the reasonings of the Apostles with these disbelievers. On the contrary, they constantly affirm the fact, that Christ did die the accursed death of the cross; and that "by the hands of wicked men he was crucified and slain."

Now, it is to prove this great fundamental truth, that Christ's death was caused immediately by the sufferings he underwent upon the cross for man's sins, that the work under our review was written. It is a pure physiological question; and we may form our opinion from the facts before us, just as we would in any other case of sudden and violent death. Christ's nature was human. In body and soul he was made in all points like any other man. This is a great fact, which should never be lost sight of when reasoning upon this subject. It was a man who went forth to encounter all the trials which were prepared for him. Christ's human nature alone suffered, his Godhead could not suffer, neither did it support or comfort the manhood under the rod. The same flesh as ours, with its beautiful and delicate network of nerves, with its fine and sensitive perceptions, was his. He could suffer to its full extent both mental and bodily pain. He was not shrouded by any superhuman agency from the keen shafts which were directed against him; he came to suffer, and though he could have had the help of legions of angels to guard him, and to rescue him from the hands of his enemies, yet because it was the will of God that he should suffer, and because he came expressly to do that will, he drank to the last drop the bitter cup of bodily pain and mental anguish which his Father gave into his hand.

As Christ's nature was that of man's, governed by the same physical laws which govern our organization, we are fully justified in reasoning from analogy on his states and habits of mind and body, living or dying, and this is all that is now attempted in the work before us.

The punishment of crucifixion was seldom or never followed by a rapid death. There was nothing but the pain and exposure to cause exhaustion and death, and these were not sufficient, even in a weak and delicate frame, to produce that result quickly. On this point, Dr. Stroud observes :—

> "The bodily sufferings attending this punishment were doubtless great, but, either through ignorance or design, have been much exaggerated. The insertion of the cross into its hole or socket, when the criminal was previously attached to it, did not necessarily produce the violent concussion which has been supposed; and as the body rested on a bar, it did not bear with its whole weight on the perforated extremities. At all events, there have been many examples of persons enduring these sufferings with the utmost fortitude, and almost without a complaint, until relieved from them by death. A fact of importance to be known, but which has not been sufficiently regarded, is, that crucifixion was a very lingering punishment, and proved fatal, not so much by loss of blood—since the wounds in the hands and feet did not lacerate any large vessel, and were nearly closed by the nails which produced them—as by the slow process of nervous irritation and exhaustion. This would of course be liable to variety, depending upon differences of age, sex, constitution, and other circumstances; but for persons to live two or more days on the cross was a common occurrence, and there are even instances of some who, having been taken down in time and carefully treated, recovered and survived. In many cases death was partly induced by hunger and thirst, the vicissitudes of heat and cold, or the attacks of ravenous birds and beasts; and in others, was designedly accelerated by burning, stoning, suffocation, breaking the bones, or piercing the vital organs."

In proof of the lingering nature of crucifixion, our author adduces many interesting facts, a few of which we may here cite, as worthy of attention. In his elaborate work, entitled "The Cross Triumphant," Bosius recites, from the "Roman Martyrology," the crucifixion of the apostle Andrew, who is said to have lived on the cross two days, which he spent in preaching and instructing the people; also that of Victor, Bishop of Amiterna, who, although crucified with his head downward, a posture unfavourable to the continuance of life, survived, in like manner, two days; which, according to Origen and other early Fathers, seems to have been the usual period during which crucified persons survived, when their death was not hastened by additional means. He likewise repeats the marvellous story of Timotheus and Maura, a married pair, who suffered in the Thebaid about the year 286, under the Diocletian persecution. After enduring many horrible tortures with invincible constancy, these pious persons were, it is said, crucified together; and, having hung alive on the cross nine days and nights, mutually exhorting and confirming each other in the faith, expired on the tenth day. The Rev. Alban Butler relates a similar case. The same year, 286, proved fatal to Marcus and Marcellianus, twin brothers, of an illustrious family in Rome, who were condemned to be bound to two pillars, with their feet nailed to the same. In this posture they remained a day and a night, and on the following day were stabbed with lances, and so died. In the year 297, by order of the Emperor Maximian, seven christians at Samosata were subjected to long and various tortures, and ultimately crucified. Hipparchus, a venerable old man, died on the cross in a short time. James, Romanus and Lollianus expired the next day, being stabbed by the soldiers whilst they hung on their crosses. Philotheus, Habibus, and Paragrus, were taken down from their crosses whilst they were still living. The Emperor being informed that they were yet alive, commanded huge nails to be driven into their heads, by which they were at length dispatched.

Thus it appears from the nature and history of crucifixion, that it is, in the generality of cases, a lingering punishment, and produces death by nervous irritation and exhaustion; but our Lord's death was not long delayed after he was nailed to the cross. A few hours brought his sacrifice to its fatal termination; and when it was necessary to end the sufferings of the thieves by breaking their legs, Christ was found dead already; to prove which, or from mere wanton cruelty, a soldier pierced his side with a spear. Now, that Christ should have so soon expired, is the more remarkable when we consider that he was in the very prime of life, and that he had had no long and wasting disease or imprisonment to exhaust his strength or debilitate his constitution. Besides, there was nothing in the previous circumstances of his persecution to account for the fact, and, with the exception of the scourging and insults which he received, he went to die with but little prospect from his bodily state of a speedy termination of his sufferings.

The occurrence of death at so early a period of his crucifixion, and the suddenness with which at last it seems to have supervened, has given rise to many conjectures; and good men have exhausted their fancies in devising reasons and framing hypotheses to account for the facts. Tertullian says, "Christ when crucified spontaneously dismissed his spirit with a word, thus preventing the office of the executioner." Origen says, "Since those crucified persons who are not stabbed suffer greater torment, and survive in great pain sometimes the whole of the following night, and even the whole of the next day; and since Jesus was not stabbed, and his enemies hoped that by his hanging long upon the cross he would suffer the greater torment, he prayed to the Father and was heard, and, as soon as he had called, was taken to the Father; or else, as one who had the power of laying down his life, he laid it down when he chose. This prodigy astonished the centurion, who said, 'Truly this man was the son of God,' for it was a miracle that he, who would otherwise, perhaps, have survived two days on the cross, according to the custom of those who are crucified but not stabbed, should have been taken up after three hours, so that his death seems to have happened by the favour of God, and rather through the merit of his own prayer than through the violence of the cross." In like manner all the fathers explain the circumstance, as the result of a miraculous interposition, or the act of that Divine power with which he was furnished. He lay down his life, say they, by his own power; he expired not by compulsion, but voluntarily, this being the signification, it was supposed, of his words, "Father, into thy hands I commit my spirit;" and, "therefore," as says Theophylact, "with a loud voice he called on death, which dared not to come to him without being called."

Modern divines have almost universally followed the fathers in the opinion that a superhuman or miraculous interposition of power was manifest in the death of our Lord. Calvin says, "that his rapid death shows an extraordinary operation of Divine providence." Grotius observes, "that his death was accelerated by Divine counsel before the failure of his natural strength, otherwise it could not have been expected to occur so soon." And Lightfoot tells us, that "he let go his soul and delivered it up into the hands of God."

These quotations are sufficient to show what the prevailing opinion always has been on the question of Christ's rapid death. All refer it to supernatural agency; and none view it as caused immediately by the execution which was done upon him. And yet we are everywhere told in the New Testament that Christ was slain by man, and died the *death of the cross.* He himself signified the kind of death he should die, when he spake of being lifted up. Paul says, "He was obedient unto death, even the death of the cross." The Jews are charged by St. Stephen with his murder; and St. Peter's words to the Sanhedrim are these, "The God of our fathers raised from the dead Jesus *whom ye slew.*"

Here, then, we are told expressly that Christ was slain by man, but if he died by his own voluntary act, or if a supernatural power were put forth to separate his soul from the body, then we cannot affirm that the Jews slew him, or that he died the death of the cross. If a criminal is ordered to be hanged, and to save him from protracted torments he is stabbed to the heart, then it cannot be said that his sentence is executed; and no more can it be said of Christ that he was crucified and slain by man, unless it can be shown that his death was immediately caused by the violence done to his body.

Then if our Lord's death was not the result of supernatural agency, but such a death as would *cæteris paribus* take place in any other man, we may as physiologists inquire into its proximate cause, and especially endeavour to ascertain the reason of its remarkable supervention.

In the execution of Christ, there were two causes of exhaustion in operation, each of which would have been sufficient to produce death, but the combination of which will alone account for its early and sudden accession. Superadded to the shock which the nervous system must have sustained by the torture incidental to this description of punishment, and which would alone in time have brought those sufferings to an end, there was in our Lord's case an overwhelming weight of deep-toned anguish upon his heart—an agony of mind which might earlier have caused his death, had he not been supported by supernatural means. This conflict of soul commenced in the garden of Gethsemane, to which he went with his disciples on the night of his betrayal. There, was his spirit sorrowful even unto death! there, in the hard struggle with his strong emotions, he fell upon the ground, and did sweat great drops of blood! there, when he tasted the bitterness of those woes which he must suffer—the weight of man's sins—the just indignation of God—the dreadful conflict, as it were, with heaven, earth, and hell—he was constrained to cry, "Father, if it be possible, let this cup pass from me!"

Now, what is the effect of strong exciting passions, and great mental emotion upon the animal functions and vital organs? Let any one answer; for it is a question to which every man who has paid the least attention to the phenomena of the passions is competent to reply. Look at a person under the influence of anger, shame, reproach, or despair, and see what a disturbance is produced in his whole system by these emotions;—the heart palpitates violently—the blood rushes to the surface—the body is swollen—the countenance becomes turgid with blood, and the whole frame is agitated by the commotion within. And these effects of great mental excitement, physiologists tell us, when aggravated, will produce death, by causing the rupture of the heart, or some great vessel in the brain or lungs. On this point, our author says:—

> "Provided they are sufficiently strong or long continued, passions of either class (the exciting or depressing) may induce death, either by simple exhaustion of vital power, or by some special injury to the heart, brain, or lungs. Agony, or the conflict between two exciting passions having opposite objects, is in this respect peculiarly efficacious; and when intense, produces violent palpitations, bloody sweat, oppression of the chest, loud cries, and ultimately rupture of the heart. Baron Haller, the father of modern physiology, observes, that excessive grief occasions palpitations, and sometimes sudden death; that the corporeal effects of anger and terror are nearly alike, including increased strength, and violent motions both in the heart and throughout the body, producing bloody sweats, and other kinds of hæmorrhage."

"Anger," says Senac, "has in certain cases torn the fibres of the heart, and even opened the ventricles."

"If any one," remarks Corvisart, "can seriously deny, or even doubt the fatal influence of the passions on the heart, let it suffice him to know that a fit of anger may produce rupture of the heart, and cause sudden death."

If, then, the effects of violent mental emotion, and strong conflicting passions, are such as here described, we cannot be surprised at the circumstances attending our Lord's last trials. Was there ever any sorrow like unto his sorrow? The weight of a world's iniquities was upon him, and the dread punishment, which would have fallen upon all the human race, was concentrated upon his head. It was not bodily pain which wrung from him the prayer for deliverance, but the deep agony and intense wretchedness of his soul. In the garden of Gethsemane this conflict commenced, and as it went on within, great must have been the disturbance of all his physical powers. There must have been all the common effects of deep sorrow and sudden horror, but greatly aggravated, as violent contractions of the heart, withir regular action; fulness of the superficial vessels, terminating in a sweat of blood; great oppression of the breathing, and a consequent disturbance of the functions of all the vital organs; and then, when added to all the mental anguish which he suffered, there was the pain of an ignominious punishment, we cannot be surprised, knowing, as we well do, the effect of such combined anguish upon the nervous and sanguineous systems, that death should have come sooner to his relief than to that of his fellow-sufferers.

That the immediate physical cause of death was a rupture of the heart is most probable, for it is by no means an uncommon result of intense mental emotion, that its strong walls should yield to the spasmodic contractions which take place under such circumstances; and when this organ is ruptured, blood is poured out into the cavity of the surrounding membraneous sac, or pericardium, and death, from the cessation of its action, takes place almost immediately.

Now, our Lord's death was marked by certain phenomena, which can be accounted for in no other way, than by supposing the heart to have been suddenly ruptured in one of those violent contractions induced by his agony of mind. His life was not protracted to the usual period for the termination of the bodily sufferings of crucified criminals—his death was sudden. A short time before, he, in a loud voice, addressed his disciple John, and prayed to his Father, which shows that it was preceded by no exhaustion sufficient to account for its supervention. He had just time enough to commit his departing spirit to God, and in a moment was gone.

After his death, a soldier pierced his side, and there flowed from the wound blood and water. Now, supposing that the pericardium had been pierced by the spear, we should, in a case of rupture of the heart, anticipate such a phenomenon. The blood, poured out from the heart into that sac or bag, would first coagulate, then separate into its two constituent parts—the solid red crassamentum, and the serum. This separation takes place very soon after blood is abstracted from a vessel, and must have been accomplished in the time which intervened between the death of Christ and the puncture of his side by the soldier;—hence, when that puncture was made, there flowed from the wound a quantity of serum, mixed with red particles of blood, which would have the appearance, to ordinary spectators, of blood and water.

Such are the grounds upon which the argument rests, and we must now leave it, with the hope that what has been said will lead to an examination of the work to which we have alluded, where a fuller investigation of the subject will be found than it is possible to bestow upon it in a limited review. The question here discussed must be interesting both to the theologian and the psychologist; to the former, as confirming the great fundamental truth of religion, and to the latter, as affording an illustrious instance of the action of mind upon body, and the effect of strong mental emotions upon the physical frame-work of the soul. That there is such an intimate connexion between the soul and body, is clearly proved by the reciprocal actions of each upon the other; but what is the connecting link, and how a spiritual essence can combine with gross matter, is one of the difficulties which has puzzled metaphysicians from the time of Aristotle to the present, and which, we believe, will continue a *questio vexata* to the end of time.

